# What are we talking about when we are talking about the audience? Exploring the concept of audience in science communication research and education

**DOI:** 10.1177/09636625241280349

**Published:** 2024-10-16

**Authors:** Ella McCarthy, Will J Grant

**Affiliations:** The Australian National University, Australia

**Keywords:** audience, science communication models, science communication theory

## Abstract

The concept of ‘audience’ is central to research and practice in science communication. When asked by a scientist for help communicating their work, who among us has not responded with the time honoured question ‘who is your audience?’ Yet what we mean when we talk about audience is not always clear: implied and ambiguous, rather than explicit and precise. This article explores this ambiguity, drawing on a systematic review of 1360 science communication research articles and a survey of 45 science communication educators. We report 10 different conceptualisations, in three groups. *Being* conceptualisations include ‘Demographic’, ‘Knowledge’, ‘Values’ and ‘Embodied’; *Doing* conceptualisations include ‘Interaction’ and ‘Dynamic’. In *Qualifiers*, we found ‘Diverse’, ‘Potential’, ‘Plural’ and ‘General’ conceptualisations. These data allow tracking of how we have conceptualised audience over time, an understanding of the groups systematically under-serviced, and a pathway to a richer discussion of this key concept for our field.

## 1. Introduction

The concept of ‘audience’ is central to both the research discipline and practice of science communication. As leading science communication scholars have long argued, in order to communicate effectively, science communicators need to understand their audience. Stocklmayer (2001), for example, has stressed that ‘science communicators who think only of the message and not of the “audience” are likely to fail’ (p. 3). Fähnrich et al. (2023: 612) have echoed this, arguing that ‘If audiences don’t pay attention to something, it kind of doesn’t exist’. Longnecker and Gondwe (2014: 144) have argued that ‘science communicators . . . must know about their target audience’. In reverse, a variety of scholars have argued that the degree to which an audience is engaged by a moment of science communication can be a key indicator of the quality of that communication (Olesk et al., 2021).

And of course, science communication researchers, educators and practitioners have an extensive history of both talking and thinking about audience. If you were to ask any of us to define an audience for a science communication product – or to articulate what is meant by the concept of ‘audience’ in general – you would of course receive a rich and engaging discussion.

But this extensive practical knowledge about audience is not necessarily supported by a concrete theoretical literature. Bucchi and Trench (2021), for example, have argued that ‘audience conceptions in science communication are often under-defined and remain implicit rather than explicit’ and that ‘audience conceptions vary considerably’ (p. 291). The *International Science Council* (2022), in parallel, has made note of the ‘systemic under-conceptualisation of “audiences” and “publics”’. Efforts should be made, Bucchi and Trench (2021) note, to ‘to describe . . . audiences further’ (p. 300) in their plurality.

At heart, what we mean when we talk about audience is not always as clear as we might think: implied rather than explicit; ambiguous rather than precise.

In this article, we seek to take up this challenge and explore this ambiguity, documenting and unpacking the ways science communication researchers and educators have conceptualised the concept of audience.

To do this, we present below two related studies. The first is a systematic review exploring the prevalence, patterns and spread of conceptions of audience in the science communication literature. The second is an online survey, exploring how science communication educators have taught students about the concept of audience.

We believe a richer understanding of what we mean when we are talking about audience offers three key benefits to our field.

First, such an understanding can allow an examination of transitions in our field over time. How are we as a community tracking against some of the key arguments leading scholars have made since the foundational debates in the 1980s and 1990s?

Second, such an understanding can allow not only an examination of who our audience is, but also – as per Dawson (2018) and Humm et al. (2020) – who our audience is not.

Finally, we hope that the picture offered here represents a useful moment in our conversation about audience, when we can move away from Bucchi and Trench’s ‘systemic under-conceptualisation’ and towards the rich picture we need to understand the people we might work with, and how we might talk about science with them.

## 2. Study 1: How do we talk about audience in the science communication literature?

Our first study is a systematic review exploring the prevalence, patterns and spread of conceptions of audience in the science communication literature.

### Methods

#### Search and collection

Our first step was the collection of a large corpus of articles from the peer-reviewed science communication literature to have raised or discussed the concept of audience.

Articles were drawn from the following five key science communication journals: *Journal of Science Communication, Science Communication, Public Understanding of Science, Research for All*, and *Frontiers in Communication: Science and Environmental Communication*.^1^

Articles were then included if the word ‘audience’ (including ‘audiences’) was used anywhere in the article’s title, abstract, keywords or body text. Search was carried out using the journals’ individual online databases,^2^ including all articles published from journal inception to completion of data collection on 8 March 2023. In total, 1406 articles were sourced at this stage, and imported for further processing into Covidence (covidence.org), an application designed for processing systematic reviews.

Articles were then excluded if the word ‘audience’ was not used in the body of the text (typically this meant it was just used in the metadata, reference list or author profile), and duplicates were removed. This resulted in a final total of 1360 papers included in the study, representing an estimated 22% of the published articles in these journals. Across the five journals examined in this study, the most included research articles came from *Public Understanding of Science* (n = 630, 25% of an estimated 2560 articles published since 1992), followed by *Science Communication* (n = 437, 30% of an estimated 1650 articles published since 1979), *Journal of Science Communication* (n = 175, 15% of 1176 articles published since 2002), *Frontiers in Communication: Science and Environmental Communication* (n = 47, 7% of 285 articles published since 2007) and *Research for All* (n = 21, 11% of 177 articles since 2017).

From this, individual usages of the term ‘audience’ were then collected from each article, alongside their surrounding textual context.^3^ If an article used the word multiple times, the clearest (usually an explicit definitional sentence) was collected; if an article used the term multiple times in distinct or contradictory ways, all relevant instances were collected. A range of other relevant data about each paper was also collected, such as type of study reported on in the paper.

#### Code development

Following data collection we commenced an iterative process of code development to categorise the ways the term audience was used. To begin, a selection of usages of the term ‘audience’ were collected, and assigned preliminary codes to indicate the contextual meaning of the term. Two coders then coded a random selection of usages of the term following the preliminary coding guide. Differences were discussed and codes revised until coders achieved over 90% intercoder agreement. The final codes are explored below in the ‘Results’ section, and our coding guide is available as Supplemental Material.

All usages of the term in the dataset were then coded according to the coding guide. Codes were not applied mutually exclusively.

### Results

We now turn to an analysis of results from the systematic literature search, exploring the prevalence, patterns and spread of conceptions of audience in the science communication literature.

#### The dataset

**Figure 1.**
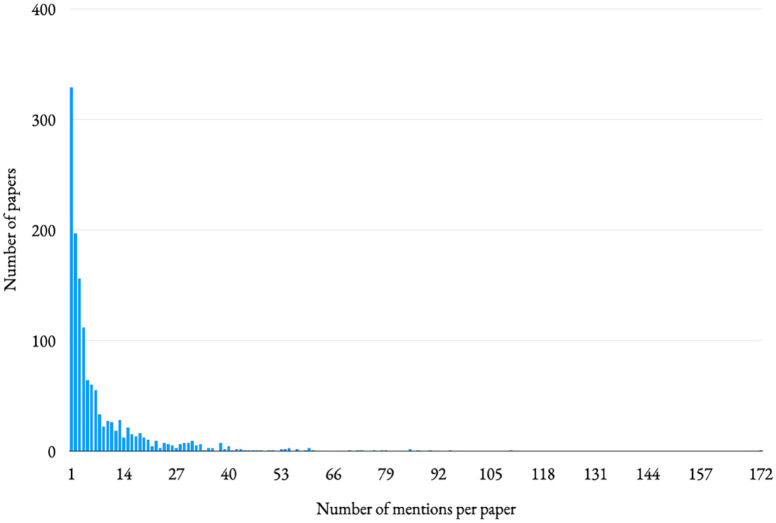
Number of papers with x mentions of the term ‘audience’. It is difficult to portray in a linear scale, but there are tiny marks up there at 95, 110 and 172 mentions.

By definition, each of the 1360 papers analysed mentioned ‘audience(s)’ at least once, with an average of 8.27 mentions per paper, a median of three mentions and a mode of one mention. The range for mentions was one mention to 172 mentions. (We might beat that maximum.)^4^ This represents (as might be expected), an extremely skewed dataset; just 10% of the papers mentioned ‘audience(s)’ more than 20 times; 1% mentioned it more than 59 times (see Figure 1). Clearly, while the term is a key part of the science communication literature, we discuss it to different extents.

Confirming Bucchi and Trench’s (2021: 291) argument that conceptions of audience in our field remain under-defined and implicit, just 4% of papers (n = 51) offered an explicit definition of ‘audience(s)’, inclusive of a definition of the paper’s or the research project’s ‘audience(s)’; to state this in reverse, some 96% of papers (n = 1309) did not include a definition of audience(s) in their writing.

#### Conceptions of audience

Following the process of iterative code development described earlier, we found 10 different ways science communication researchers have conceptualised audience, in three broad categories. The three broad categories we have collected as *Being* (things about the audience themselves), *Doing* (things an audience is doing) and *Qualifiers* (modifications to the previous). It should be noted that these categories are not rigidly mutually exclusive in either our categorisation or the original authors’ usages.^5^ Instead, they offer ways to give meaning to the ways science communication researchers have thought about audience.

First, within the *Being* category, we identified ‘Demographic’, ‘Knowledge’, ‘Values’ and ‘Embodied’ conceptualisations, an expansion on the typology proposed by Hill et al. (2022). Within the *Doing* category, we identified ‘Interaction’ and ‘Dynamic’ conceptualisations. Within the *Qualifiers*, we identified ‘Diverse’, ‘Potential’, ‘Plural’ and ‘General’ conceptualisations. To begin, in Table 1, we outline the total numbers in each category. Below, we explore the ways these categories manifested with examples.

**Table 1.** Overall number of articles (total n = 1360)/proportion of articles to have used each conceptualisation of audience.

Audience conceptual category		Number of articles	Proportion as %
Being	Demographic	433	32
	Knowledge	376	28
	Embodied	244	18
	Values	88	6
Doing	Interaction	1038	76
	Dynamic	335	25
Qualifiers	Diverse	132	9
	Future/potential	60	4
	Plural	910	67
	General	652	48

#### Overall

Table 1 presents the overall numbers and proportions of articles we found in each category.

#### Audience as being

##### Demographic conceptions

A large number of articles in our dataset (n = 433, 32%) conceived of audience in what can be labelled ‘demographic’ terms, using statistically measurable, putatively objective^6^ criteria such as age, nationality or gender:

. . . children . . . as the user audience

. . . the characteristics of the audience who were the focus of the paper (for example women, immigrants)

. . . the benefits of scientific research to the Taiwanese audience

While 433 articles conceived of ‘audience’ through demographic categories, many drew on multiple demographic elements simultaneously, leading to a total of 582 demographic conceptions. Table 2 shows the number of articles using particular demographic categories. As can be seen, we clearly have a long history of considering communication with audiences based on occupation, age or education (as examples), but a rather thin literature exploring science communication with audiences with disabilities.^7^

**Table 2.** Numbers of papers using particular demographic categories.

Demographic category	Number of articles
Geographic location^a^	129
Occupation	127
Age	106
Education	58
Gender	30
Class	23
Political affiliation	22
Race/ethnicity/cultural background	16
Religious affiliation	10
Languages spoken	9
Family makeup/household composition	8
Citizenship status	6
Generation	4
Disability status	3
Marital status	2
Appearance	1
Vaccine status	1
Incarnation status	1
‘General demographics’	23

a We count geographic location as both a demographic and embodied conceptualisation, as discussed below.

##### Knowledge conceptions

A similarly large slice of our dataset (n = 376, 28%) conceived of audience in relation to knowledge they did or did not hold:^8^

. . . the framing, simplifying, or popularizing of scientific results for nonexpert audiences

. . . part of the audience whose understanding of science, and more specifically of science in society, needs updating?

. . . it may be safer to assume the audience knows nothing or very little . . .

##### Embodied conceptions

A slightly smaller number of articles in our set (n = 244, 18%) conceived of audience in terms of how they were physically embodied or located in space. Of these 129 were geographic location (also considered as a demographic category above), while 115 pointed to other embodied conceptualisations:

. . . a packed audience at Carnegie Hall.

. . . a concrete audience (a totality that is bounded by an event or physical space)

. . . viewing on small screens encourages the production of brief online videos

##### Values conceptions

Finally, a small number of articles (n = 88, 6%) conceived of audience in terms of values: beliefs regarding what are good for themselves or society at large. These were either explicitly held and known by the authors, or assumed:

. . . deep sense among audiences that science is important, and that science should be valued.

. . . If an audience does not believe that a speaker has their interests at heart (goodwill), does not share their sense of values (goodness)

. . . improving climate change acceptance should involve careful consideration of the target audience’s values

#### Audience as doing

##### Interaction conceptions

The largest category in our dataset (n = 1038, 76%) were articles talking of an audience as interacting with a piece of science communication content. This is, of course, perhaps unsurprising – the roots of the word audience can be found in the Latin *audire*, to hear:

. . . audiences may have different motivations for seeking out media content

. . . because the journal is open-access, anyone (policy makers, laypeople, activists, etc.) can view it

. . . when there is an audience watching

. . . at the pop up stalls . . . we engaged with a diverse audience

##### Dynamic conceptions

Meanwhile, a smaller (but still substantial) group of articles (n = 335, 25%) conceived of audience as dynamic – not only interacting with content, but actively involved in co-constructing meaning, or changing with the communication:

. . .an awareness of the audience as participants in the construction of meaning.

. . . online platforms and crowds could potentially play a role on both sides of the process, by lowering entry barriers on the supply and by allowing audience preferences to independently express themselves on the demand side, with less centralized gatekeeping.

. . . audiences are not merely passive receivers of risk information, but have the capacity and inclination to engage actively in interrogation of risk communications and other related risk information.

**Figure 2.**
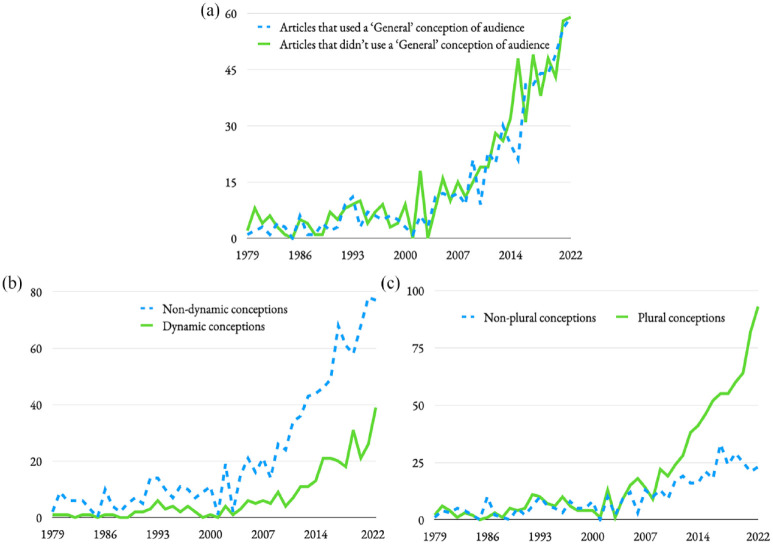
Trends in audience conceptualisation over time. Clockwise from left: a) Articles that talked of a ‘general’ audience (blue dashed), compared with articles that did not use this framing of audience (green), b) plural (green) versus non-plural (blue dashed) conceptions of audience over time and c) dynamic (green) versus non-dynamic conceptions (blue dashed) of audience over time.

Figure 2c below charts the usage of dynamic and non-dynamic conceptions of audience over time. While we clearly cannot point to a transition towards participatory, involved, *dynamic* conceptualisations of audience (as compared with non-dynamic ‘audience as receiver’ conceptualisations), we can suggest a growth in such usages over time, particularly over the last decade.

#### Qualifiers

Our final broad category of audience conceptions include a range of qualifiers to the above *Being* or *Doing* conceptions; or more broadly, ways of talking about an audience that avoided rich specificity.

##### Diverse conceptions

A range of articles (n = 132, 9%) conceived of audience as potentially diverse, either meaning a variety of different people were in a room, or the audience were somehow outside what might be considered ‘normal’ for that type of event:

. . . this participant strove to incorporate ‘loads of different emotions’ to engage as diverse an audience as possible.

. . . most trainings are not designed to account for diversity, nor are they specifically designed to help scientists engage with diverse audiences.

. . . reaching a diverse audience that include deaf sign language users, oralized deaf people and hearing language learners

. . . a culturally and spatially diverse audience was selected, which featured participants from a range of sociodemographic backgrounds, social groupings, ages, and nationalities.

##### Future/potential conceptions

A small number of articles (n = 60, 4%) conceived of an audience as appearing at a future point in time, and/or a hypothetical potential audience:

. . . while producing their film, students were in a social-bubble, continuously assessing whether their descriptions and depictions would be understood by their audience.

. . . the potential audience for the media coverage runs into many millions of readers, viewers, and listeners’

. . . though some addressed a broader potential audience, with a range of strategies and degrees of success.

##### Conceptions of plural ‘audiences’

A large component of the dataset (n = 910, 67%) conceived of ‘audience’ as somehow plural. This could include multiple discrete audiences (the attendees at multiple science communication events), or the simple recognition that those engaging with science communication should rarely be treated as a singular cohesive group: (Note, this conception differs from an article offering multiple conceptions of audience.)

. . . shares first-person stories about science with public audiences

. . . that is why COVID-19 led the audiences to reconnect

. . . mass media discourses of emerging sciences and technologies have been analyzed in comparison with public discourses shared by mass audiences.

Figure 2b below illustrates the use of plural and non-plural conceptions of audience over time, with a clear transition over the last decade or so towards plural conceptions.

##### General conceptions of ‘audience’

Perhaps concerningly, almost half of the sample (n = 652, 48%) conceived of audience as general, non-specified, mass, broad and/or simply ‘public’:

. . . any content that is appropriate for a general audience . . .

. . . conveying complex scientific narratives to a broad audience has been an ever-present challenge for science communicators and educators.

. . . the creators most well-known to mass audiences, behind the most popular movies and shows

While, of course, we in science communication should consider how we engage with various audiences, including audiences outside the traditional scientific sector, we say ‘perhaps concerningly’ regarding this conceptualisation because this category reflects somewhat of a simplistic view of audience that hopefully we are moving away from. Figure 2a suggests this hope might be misplaced.

## 3. Study 2: How we teach the concept of audience

We can now turn to the second component of this investigation, exploring how science communication educators have taught students about the concept of audience.

### Methods

#### Data collection

Data for this study were collected through an online survey, sent out through email to a global sample of science communication tertiary educators. The sample of participants was selected from the full list of speakers and chairs from the 2023 *Public Communication and Science Technology Conference* (n = 456), filtered by biographical information indicating a teaching role at a tertiary level educational institute, resulting in a total of 123 invitees.

The survey was hosted on the online survey platform Qualtrics. After simple categorical inclusion questions (‘Do you teach Science Communication (or a similar related topic) at the tertiary education level’, ‘Do you teach your students about audience/audiences’), the survey then asked a series of questions about audience. Two were open text questions (‘How do you teach your students about audience/audiences’, ‘How do you define audience/audiences in your teaching’), and two sought to gauge timing (‘At what stage(s) do you teach your students about audience(s)?’) and importance (‘How relevant is the concept of audience to your teaching?’) None of the questions were mandatory, meaning there is some variation in the number of responses per question. Our survey questions are included in Supplemental Material.

Written data from the open text questions was then coded using the same coding as used above.

This study was approved by our University’s Human Research Ethics Committee (Protocol 2023/313).

### Results

#### Importance and timing

Of the 123 people invited to participate, 45 surveys were completed, a response rate of 37%. Of these, 40 agreed that they taught Science Communication (or a related topic) at the tertiary education level, and 36 taught their students about audience/audiences. All of those who answered the question on importance (n = 25) considered it important: either ‘Very Relevant’ (n = 19, 76%) or ‘Quite Relevant’ (n = 6, 24%) to their teaching. Responses on timing were more varied. Here, the 25 respondents selected 56 timing options (multiple selections were possible): 14 ‘at the start of my teaching’, 20 ‘early in my teaching’, 13 ‘mid-way through my teaching’, 5 ‘towards the end of my teaching’, and 4 ‘at the end of my teachings’.

#### Defining audience

When asked how they defined the concept of audience, respondents (n = 23) offered a variety of rich responses. First, some noted a caution in providing precise definitions to their students:

I do not present the students with an actual definition of audience/audiences . . . they have to define their target audiences themselves.

I don’t have a specific definition.

Nevertheless, many offered definitions we could code according to the schema presented above (of *Being, Doing* and *Qualifiers*), often weaving a few different conceptions in their definition.

#### Audience as being

Of the *Being* group of categories, we found demographic articulations:

We talk about the concept of audience as just different groups of people . . . university students, your lab group, K-12 students, public audiences (which further break down into a number of different audience types), etc.

Embodied articulations:

My focus is digital audiences and so I teach from the perspective of digital and platform publics, so focusing on digital platform affordances . . . I work with students to gather information about particular audiences (age, gender, location, attitudes, values, lifestyle) . . .

Knowledge based articulations:

As knowledgeable persons, maybe not always scientifically knowledgeable about the technology or science at stake in the communication, but very likely experienced with the influence or impact of the science / technology at stake in the communication.

Values based articulations:

I then ask: Who are they? Why are they listening/reading/participating? Why do they care?

I describe three types of audiences -interested audience, uninterested audience, and skeptical audience.

Groups of people with different goals and interests

#### Audience as doing

Among the ‘doing’ group of conceptions, we found a range of interaction based definitions:

I mostly categorize them accordingly to the situation = listeners for podcasts, readers for articles, participants for co-creation processes.

In general I refer to audience with different wordings depending on the context (communication situation): participants, listeners, public, co-creators, stakeholders, actors, group, public, etc.

. . . In several ways, depending on the nature of the communicative act. Broadly speaking there are those in receipt of information, those in dialogue or exchange of information . . .

And dynamic articulations:

As active co-constructors of knowledge and or the content that is being communicated.

Those where action of some form is involved.

#### Qualifiers

We also found a range of the qualifiers noted earlier, though perhaps somewhat attenuated compared with the literature discussed earlier. We found those stressing audiences were both diverse and plural:

As highly plural /diverse.

And others stressing that ‘general’ framings of audience were conceptually weak:

There is no ‘general public’.

#### Overall

Overall, science communication educators used similar framings – at a similar rate – as we found in the literature. As with the literature, the most common conceptualisations were interaction based (74% of educators, 76% of definitions from the literature), and many stressed the plurality of audiences (65% of educators, 67% of definitions from the literature). Happily, ‘general’ framings were less common (22% of educators vs 48% of definitions from the literature). Compare Table 1 above and Table 3.

**Table 3.** Frequency of conceptions of audience offered by science communication educators.

Audience conceptual category		Proportion as %
Being	Demographic	22
	Knowledge	35
	Embodied	17
	Values	4
Doing	Interaction	74
	Dynamic	22
Qualifiers	Diverse	4
	Future/Potential	0
	Plural	65
	General	22

### How do educators teach the concept of audience?

So, how then do science communication educators teach their students about audience?

#### Stressing centrality

First, it is worth noting that when asked this question, a number of respondents took the chance (building on the explicit check box question discussed earlier) to stress the centrality of audience to their teaching:

It’s central to almost everything.

We run an MSc in science communication and ‘audiences’ are embedded throughout our core and optional modules

Know your audience is a core tenet of science communication. For communication to be effective then communicators need to know who they are talking to. What do they already know about the topic, what do they need to know? What do they value, believe? This flows through all course materials as my students tend to create materials for an audience they must define.

#### Recognising plurality

Building on this, many of the educators then stressed it was essential to teach students about the plurality of audiences – that a singular audience was anathema to good science communication:

Consider different audiences

I teach our students to be aware that there are many different audiences and that you need to think well about which subgroup you are working with, what their needs are and whether this fits your goal.

On target audiences . . . address epistemological differences between, e.g., journalists, policy-makers, scientists and ‘citizens’

The inherently diverse nature of audiences, and the importance of segmenting audiences, picking relevant audiences according to strategic communication goals

#### Drawing on theory and literature

Educators then offered a key role for literature and theory in helping students to understand audience. While some were more general here, some offered direction towards particular sources:

Supporting course resource information outlines how to identify an audience, what is important when considering how to communicate with different audiences

With the help of various science communication and psychology theories . . .

I first have them read some background material about knowing your audience . . .

The concept of audiences is covered in quite a few ways, by inclusion in lectures, examining case studies and real life examples (for example when thinking about concepts like audience segmentation) and by reading and analysing, with discussion, journal articles and book chapters that include audiences as concepts.

A combination of lecture-style materials, combined with readings of third party material.

I cover some theoretical aspects about how we might conceptualise audiences (from Michael’s PiGs and PiPs (Michael, 2009) to distinctions between audience, public, participants etc).

I have them read a book chapter about audiences (Longnecker, 2023). I present lecture material about audiences, e.g. looking at motivations and barriers for informal learning opportunities, including John Falk’s (2006, 2009) visitor identities.

#### Practical activities

Finally, educators offered a range of practical activities through which they sought to facilitate students’ learning about audience including roleplaying and theatre games and the development of research based personas:

I use some roleplaying games to give students a feel for different audiences

We use participatory theatre, games and workshops.

I have them write a short paragraph about their own research. They exchange paragraphs with people in different fields and highlight unfamiliar terms, etc. We discuss how this relates to the concept of audience

We ask them to make personas, preferably based on real / available data

I teach about the creation of research-based personas.

Students are asked to do target audience research (roughly defining the group. determining the starting position and the preliminary knowledge of the TA).

I cover some practical ways we can go about mapping out audiences from social psych to worksheets.

## 4. Discussion and conclusions

In this article, we have explored the concept of audience in science communication research and education; to address Bucchi and Trench’s (2021) argument that ‘audience conceptions in science communication are often under-defined and remain implicit rather than explicit’ (p. 291). In doing this, we confirmed Bucchi and Trench’s assertion: just 4% of the papers in our sample offered an explicit definition of either the audience they were addressing or audience in general. Nevertheless, in exploring the implicit uses of the term, we found 10 different ways science communication researchers and educators have conceptualised audience, in three broad categories. In our *Being* category, we identified ‘Demographic’, ‘Knowledge’, ‘Values’ and ‘Embodied’ conceptualisations, an expansion on the typology proposed by Hill et al. (2022). Within the *Doing* category, we identified ‘Interaction’ and ‘Dynamic’ conceptualisations. Within the *Qualifiers*, we identified ‘Diverse’, ‘Potential’, ‘Plural’ and ‘General’ conceptualisations.

We now reflect on our findings, addressing the three key benefits raised at the start of the article: examining transitions in the field of science communication over time; examining the audiences science communicators consider and the audiences we leave out; and how we move from here to a richer discussion of this key concept into the future.

### Science communication over time: Are we moving away from the deficit model?

Since the early 1990s (Wynne, 1992; see also Grant, 2023), many in the field of science communication have argued for a shift away from what is often termed ‘deficit model’ communication – communication in which scientific knowledge is afforded epistemic priority above all other forms of knowledge – towards more dialogic, participatory or co-productive forms of communication. While we cannot address the entirety of this debate, our data provide some interesting new insights on trends in our field.

First, some positive evidence: over the last decade (see Figure 2b), we have seen marked growth in the use of plural conceptions of audience, above that of singular: audience*s* rather audience. This suggests a desire by science communication researchers to avoid the totalising/generalising/reductive language that lumps all receivers of a science communication product together. A good thing!

More positive evidence: over the last decade (see Figure 2c), we have seen growth in the number of research articles to have considered their audience as dynamic: changing, participating, perhaps actively involved in co-producing knowledge. While non-dynamic conceptions remain dominant in our field (i.e. perhaps treating the audience as static or passive), the growth alone is a positive sign (see Hom et al., 2011; Reincke et al., 2020; Seethaler et al., 2019).

But perhaps more worryingly, the use of ‘general’ framings of audience (as most famously used in the phrase ‘the general public’) remains strong (see Figure 2a). It appears many in our field continue to frame their thinking of audience around the simple binary of ‘scientist’ and ‘general public’. Happily, most of the educators who responded to our survey appear to have moved on, rejecting such framings. While our respondents cannot be taken as representative of all science communication educators (perhaps those most interested in this sort of discussion responded), it is somewhat a sign of hope.

Here it is worth noting that future work in this space could explore trends in audiences conceptualised by knowledge. While it is clear that the deficit model simply takes an audience as ‘deficit’ in the particular knowledge at hand, a more positive use of knowledge conceptualisation could explore the other forms of knowledge brought to a science communication moment by any particular audience. How, for example, do science communicators engage with lawyers, or scholars from the humanities, or well-versed amateur hobbyists in a field?

### Who are we not thinking about?

Building on this, many in the field (see, for example, Canfield and Menezes, 2020; Dawson, 2019; Judd and McKinnon, 2021) have argued that much work remains to be done to build a more inclusive science communication: better recognising intersectional diversity and better addressing systemic minoritisation. Here our data offer a glimpse not only of the audiences we are considering, but also those we are not. Table 2, in particular, paints a picture of a literature paying significant attention to the demographic categories of occupation, age and education, but only moderate (or even thin) attention to gender, class and disability. Of course, attention could be being paid to these categories in other journals (e.g. discussion of science communication within disability focused journals), and it may be that those considering these issues might focus first on those journals. Nevertheless, it is clear there is a gap within the explicitly science communication oriented journals. More work in this space in the future could better draw out patterns and trends in these data.

Beyond this, it is also worth noting here that while 132 articles in our sample talked of audience as diverse or inclusive of diversity, very few (just 3% of the literature) did so in a specific manner. And so we would caution against simplistic uses of this term. At heart, saying an audience is ‘diverse’ is probably fine if the goal is to imply that there are different people in the room, but can we not add a little specificity? Are they diverse in knowledge, or values, or occupation, hearing ability or political ideology? It should be stressed here that one individual in themselves is not ‘diverse’, no matter the colour of their eyes.

### Towards a richer conversation

And so finally, we can stress once again – building on the arguments of Stocklmayer (2001), Fähnrich et al. (2023), Longnecker and Gondwe (2014) and Olesk et al. (2021) noted at the beginning of the article, and the evidence we have detailed earlier – that the concept of audience is clearly of significant importance to the field of science communication.

But we have not, as Bucchi and Trench argued, done the work to make our thinking about audience explicit.

We hope that this work furthers this conversation.

We do not envisage that our categorisation should be the end of this process. Clearly, we have not explored every paper in the broader science communication literature, or in literatures to have discussed science communication ‘from the other side’. Similarly, it should be recognised that a sentence in an article (no matter how worked over and peer-reviewed) cannot be taken as the author’s final word on the subject.

Furthermore, there might be better ways to categorise our collective thinking on audience, or there might be other, non-categorical ways to approach the problem. As one participant in Hill et al. (2022) noted, ‘I just view the audience as actual humans who have actual feelings . . . They’re just real people . . . Souls if you will’.

Beyond this, we believe more should be done to explore, document and share our practices of teaching audience. Our simple snapshot above offers only a glimpse, when perhaps textbooks could be published on the topic. Could we share methods in more detailed interviews, or through sessions at conferences, or through collected online resources?

Nevertheless, as such a key part of our collective gaze, we should do this work, and reflect more deeply about what we are talking about when we are talking about audience.

## Supplemental Material

sj-pdf-1-pus-10.1177_09636625241280349 – Supplemental material for What are we talking about when we are talking about the audience? Exploring the concept of audience in science communication research and education Supplemental material, sj-pdf-1-pus-10.1177_09636625241280349 for What are we talking about when we are talking about the audience? Exploring the concept of audience in science communication research and education by Ella McCarthy and Will J Grant in Public Understanding of Science

## Notes

1. These are, of course, not the only journals to focus on issues relevant to the field of science communication, nor the only journals to include science communication articles. Nevertheless, we hope they provide a good coverage of key thinking in the field.

2. Online journal databases: https://jcom.sissa.it/, https://journals.sagepub.com/home/scx, https://journals.sagepub.com/home/pus, https://uclpress.scienceopen.com/collection/UCL_RFA, and https://www.frontiersin.org/journals/communication/sections/science-and-environmental-communication

3. For example, the sentence The picture of a simplistic dichotomy, for instance that one group of experts had a common view on a topic that is opposed to the consensus view of another group of experts or a lay audience, is challenged by this interpretation was drawn from Allgaier (2012).

4. In total, 229. Booyah!

5. Indeed, some 1142 of the 1360 articles (84%) conceived of audience in ways aligned with multiple of the categories below.

6. We accept, of course, that many (if not all) demographic categories are complex social constructs rather than immutable facts about a person. Nevertheless, they are often treated as if they can be objectively known, so it is useful to continue that treatment here.

7. It should be stressed here that this does not mean these concepts are not considered elsewhere; for example, the engagement between science and audiences with different dis/abilities may be considered in the disability literature. We only seek to show what has been explored in what might be considered the central discussion of science communication.

8. It is worth noting that while knowledge may clearly associate with particular demographic categories (most obviously education and occupation), they can usefully be treated as conceptually distinct.
