# Supplementary material for: What are we talking about when we are talking about the audience? Exploring the concept of audience in science communication research and education
Source: Public Underst Sci. 2024 Oct 16;34(4):408–23. doi: 10.1177/09636625241280349 (PMC12038061; doi:10.1177/09636625241280349)
Supplement: sj-pdf-1-pus-10.1177_09636625241280349 – Supplemental material for What are we talking about when we are talking about the audience? Exploring the concept of audience in science communication research and education [file sj-pdf-1-pus-10.1177_09636625241280349.pdf]

Supplemental material for:
What are we talking about when we're talking about the audience? Exploring the concept of audience in science communication research and education

E. L. McCarthy & W. J. Grant

1: Coding Guide
2: Survey Questions

Coding guide for ‘What are we talking about when we’re talking about the audience? Exploring the concept of audience in science communication research and education’

Full Text Review

Search for ‘audience’ in body of text
Papers are included if they mention the term ‘audience(s)’ once or more in the body of the text.

Mentions of audience in paper
- Must be in body of text
- Not in title or abstract or keywords
- Not in the bibliography or author sections
- May include subtitles within body of the text and image/figure descriptions

Extraction via: Quality Assessment Template

Does this paper present new/research data?

Yes, it presents new/research data
No, it does not

Below are some recorded responses:
Yes – “Since a comprehensive analysis of German science videos is missing as well as a typology of films and providers, several hundreds of videos were examined, and a corpus of 400 videos was compiled.”
Yes – “A total of 159 participants submitted their answers concerning their receptiveness to the humour, demographic information and comments”
No – “As a case study, we analyze an article of the psychiatrist Henrique Roxo published in 1942 in two publications directed to different publics.”
No – “This essay examines a highly popular comic series published in Spain between 1969 and 1970 which focused on Felix Rodríguez de la Fuente (1928-1980), a prominent and influential naturalist and media icon, as main character.”

What type of data is in this paper?

Human participant data
Non human participant data
Both human and non-human participant data
Discussion paper/other

Below are some recorded responses:
Human participant data – “We recently completed a 3-year experiment to use audiences of non-scientists to evaluate the effect of training on STEM (Science, Technology, Engineering and Math) graduate students’ communication ability.”
Non-human participant data – “The comparative analysis is based on a careful reading and coding of PNW reports and opinion pieces appearing in six Canadian news outlets between September 2014 and August 2017.”
Both human and non-human participant data – “Our research involved analysis of press coverage and interviews with scientists about their experiences of, and reactions to, media”
Discussion paper/other – “This essay addresses two different kinds of reason “practical and epistemic” converging at that point.”

Is this a media analysis or event paper?

Yes, media analysis
Yes, event paper
Yes, both
No, neither

Below are some recorded responses:
Yes, media analysis – “Using American and British newspaper coverage of the autism vaccine controversy as a case study, this article takes a ‘behind the scenes’ look at normative pressures that may influence whether such information appears in coverage. In particular, can holding health officials accountable for their actions potentially ‘crowd out’ mobilizing information?”
Yes, event paper – “Events consisted of one or a panel of experts to speak to their expertise through a 30-40 minute talk followed by a question and answer session which lasted a similar amount of time. Data were collected by the first author (CO) during each of these events.”
No, neither – “"We collected a nationally representative sample of 1,554 United States residents through a Qualtrics XM survey panel during August 2020. In the Qualtrics XM panel potential respondents from across the United States sign up to participate in online surveys through the Qualtrics website"”

Does this paper have an explicit definition of audience?

Yes
No

In this category, we are coding for an explicit definition of the concept of audience rather than the explanation of their own audience.

Below are some recorded responses:

No - Interesting: They felt that putting the effort into visualizing and reaching out to an audience might restrict their ability to express themselves

No - It has a specific description for intended audience from court transcripts but not an explicit definition of audience: "Surprisingly, these courts regarded the intended audience as equal to the expert with a very specific kind of knowledge and expertise instead of lay observer.

No - explicitly saying this is not thought about very much at all - "They do not necessarily think very much about audiences, but if they do, they might have a vague idea of different target groups with an interest in their particular knowledge"

How many times is 'audience' mentioned in this paper?

-Once

- Multiple (add number in supporting text box)

In this section we are looking to determine how often 'audience(s)' appears in the body of the text. This is any section after the title & abstract and before the end of the conclusion, non-inclusive of title, abstract, authors, acknowledgements, etc.

Below are some recorded responses:

Multiple – "33"

Multiple – "2"

How is audience discussed in this paper?

-Enter a quote of the richest sentence in the box below for notes

This section is a place to record one sentence of the richest inclusion of the word 'audience(s)' from within the paper

Below are some recorded responses:

"I've found the VLT method to be effective with science conference audiences, farmers groups, university classes, and the general public."

"During the period under our investigation the prevalent term was 'popularization', i.e. simplifying the science to the wider audience."

Is audience conceived of in terms of demographics in this paper? (Inclusive of statistical data i.e. age, gender, occupation, tax income bracket)

Yes

No

This category is to record if the paper conceives of audience in terms of demographics, this could be either using demographic categories in context of audience, or conceiving of audience in a way that could be used as a demographic category.

Below are some recorded responses:

Yes - includes policy makers – occupation

Yes - roles/occupations referring to future/current/potential audiences
Yes - occupation based - teachers and students
Yes - occupation - the public, legislators, insurance entities, and other medical professionals, also optometrists
No

Is the conception of audience in this paper diverse non-specified? (E.g. the word 'diverse' is used to talk about audience)

Yes
No

This category is to record if this paper conceives of audience in a non-specific diverse way, if the word diverse is used to describe audience(s).
Below are some recorded responses:

Yes - "diverse population of audiences"
Yes - "diverse, nonspecialist audiences."
No

Is the conception of audience in this paper diverse specific? (E.g. does the paper mention diverse audience groups such as Auslan users, Women in STEM, etc. ?

Yes
No

This category is to record if this paper conceives of audience in a specific diverse way. Diverse audience groups are those outside the 'normal' for the context presented. For example Women in STEM are a diverse group in STEM, Spanish speakers are within the 'normal' group in Spain.
Below are some recorded responses:

Yes - mentions audience and presenters as being within minorities inclusive related issues such as "autism, racism and sexism in science"
Yes - "When designing the website, the usability for a non-linguistic audience was of the utmost importance" - non-linguistic users are a diverse group

Is the conception of audience in this paper dynamic? (Audience characterized by their constant change/activity/progress)

Yes
No

This category is looking to record if the paper conceives of audience as active, dynamic, and/or changing.
Below are some recorded responses:

Yes – "Those videos were evaluated by an audience
drawn from a large pool of undergraduates in a Communication course" – the audience in this paper are active

Yes – “But as has been noted by generations of media scholars, audiences are active in making their own meanings, whether they are reading a book or using biofeedback locative media.”

No

Is the conception of audience in this paper embodied? (Inclusive of physical considerations and spatial presence)

Yes

No

This category is to determine if this paper conceives of their audience in an embodied manner, this is inclusive of physical considerations and spatial considerations.

Below are some recorded responses:

Yes – "Competition from other formats, the fight or audiences’ attention and viewing on small screens encourages the production of brief online videos." - embodied nature of viewing on smaller screens

Yes – “considerations of body/hearing "reaching a diverse audience that include deaf sign language users, oralized deaf people and hearing language learners"

No

Is the conception of audience in this paper general/non-specific (Inclusive of 'lay audience', 'public', 'publics', and 'general audience'')

Yes

No

This category is to understand if the audience is conceived of in a general or non-specific way, this is not inclusive of ‘target audience’

Below are some recorded responses:

Yes - 'a mixed general audience'

Yes - "all audiences" "intended audience"

Yes - 'the audience'

Yes - 'intended audiences'

Yes - 'non technical' & 'general audience'

Yes - 'lay audiences' as only mention of the term

Yes - 'large audience' is only mention

Yes - 'target audience' & 'broad audience'

Yes - 'broader audiences’

Yes - 'general audiences'

Yes - though it does acknowledge subsections of the ' wider public' also includes 'non-technical audience'

Yes - but conceives of audience as a more broken-down idea of this, however, foundationally the same
Yes – “the public”
Yes – “nonspecialist”
No

Is the conception of audience in this paper interaction dependent? (Inclusive of how audience interacts with the work, social media, etc.)

Yes
No

This category is to understand if audience are being conceived through the means in which they interact with work/social media/a certain platform etc.
Below are some recorded responses:

Yes - "policy audiences"
Yes - a specific audience at this individual's talk, including one specific question-asking member of the audience
Yes - pp 273 "lay listeners" of popular music
Yes - yes, audiences of newspapers
Yes - corporate and government visitors - they are visiting
Yes - an audience using social media channels
No - No, audience is mentioned as general - but in the context of the media, not general for the media
Yes - inclusive of research audience and funders
No

Is the conception of audience in this paper knowledge specialized? (Inclusive of researchers, high school finishers, but not inclusive of 'lay' or 'general' audiences)

Yes
No

This category is to record if audience is being conceived in a way that it specific to the knowledge that they hold/do not hold. This is not inclusive of lay or general audiences.
Below are some recorded responses:

Yes - 'non technical'
Yes - Yes but briefly in terms of context of 'stakeholders' and that audiences may be considered in regard to their 'particular knowledge'
Yes - prior knowledge on subject involved in conception of audience
Yes - non-technical audience
Yes – “legislators, insurance entities, and other medical professionals”
Yes – “nonspecialist”
No

Is the conception of audience in this paper of a potential/future audience?

Yes

No

This category is to record if audience is conceived in this paper as being a 'potential' or 'future' audience, i.e. audience that is not yet reached. This is not inclusive of intended audience

Below are some recorded responses:

Yes - yes it explicitly states 'potential audiences' in the form of roles/occupations

Yes – "the potential audience reach of Season Spotter"

No

Is the conception of audience in this paper value driven/dependent? (Conceives of audience through what they may value)

Yes

No

This category is to determine if audience are conceived of through the values, including those that they hold/are thought to hold.

Below are some recorded responses:

Yes - "difficult to generalize to all audiences since people's values may play a more central role"

Yes - " in the context of climate change, at least six different audiences have been identified, each with their own set of beliefs, values, attitudes, and behaviors"

No

Does this paper conceive of multiple audiences? (Is audience referred to as audiences?)

Yes

No

This category is to record is the paper conceives of audience as plural, e.g. uses 'audiences' or 'multiple audiences'

Below are some recorded responses:

Yes- Yes, during both in-text body-of-text uses of the word stem 'audience' it appears in its plural form

Yes – 'audiences'

Yes - audiences are considered in plurality

Yes - 'large audiences'

Yes - in plural form and also with a plural descriptor 'myriad'

Yes - All 4 in-body-of-text references are to the plural form of audience: audiences

Yes - "multiple audiences"

No

Are there plural conceptions of audience in this paper? (As defined through the above categories)

Yes – if more than one above is selected

No

This category is to record if plural of above categories are being recorded, if the papers conceive of audience in more than one way.

Below are some recorded responses:

Yes

No

Survey questions for What are we talking about when we're talking about the audience? Exploring the concept of audience in science communication research and education

1.) Do you teach Science Communication (or a similar related topic) at the tertiary education level? [One selection allowed]

a. Yes

b. No

2.) Do you teach your students about audience/audiences? [One selection allowed]

a. Yes

b. No

3.) How do you teach your students about audience/audiences?

[Open text box response]

4.) How do you define audience/audiences in your teaching?

[Open text box response]

5.) At what stage(s) do you teach your students about 'Audience(s)'?

[multiple selections allowed]

a. At the start of my teachings

b. Early in my teachings

c. Mid-way through my teachings

d. Toward the end of my teachings

e. At the end of my teachings

6.) How relevant is the key concept of 'Audience(s)' to your teaching?

[One selection allowed]

a. Very relevant

b. Quite relevant

c. Somewhat relevant

d. Not very relevant

e. Not relevant
